# Calcium-sensing receptor acts as an antiviral factor for rotavirus infections and participates in cellular antiviral response

**DOI:** 10.22038/IJBMS.2022.64533.14201

**Published:** 2022-08

**Authors:** Haohai Huang, Dan Liao, Bin He, Yejia Cui, Rong Pu, Guanghui Zhou

**Affiliations:** 1 Department of Clinical Pharmacy, SSL Central Hospital of Dongguan, Dongguan Third People’s Hospital, Affiliated Dongguan Shilong People’s Hospital of Southern Medical University, Dongguan, Guangdong, China.; 2 Medical and Pharmacy Research Laboratory, SSL Central Hospital of Dongguan, Affiliated Dongguan Shilong People’s Hospital of Southern Medical University, Dongguan, Guangdong, China; 3 Department of Gynaecology, SSL Central Hospital of Dongguan, Dongguan Third People’s Hospital, Affiliated Dongguan Shilong People’s Hospital of Southern Medical University, Dongguan, Guangdong, China; 4 Central Laboratory, SSL Central Hospital of Dongguan, Dongguan Third People’s Hospital, Affiliated Dongguan Shilong People’s Hospital of Southern Medical University, Dongguan, Guangdong, China; 5 Department of Clinical Laboratory, SSL Central Hospital of Dongguan City, Affiliated Dongguan Shilong People’s Hospital of Southern Medical University, Dongguan, Guangdong, China; 6 Department of Rehabilitation medicine, SSL Central Hospital of Dongguan, Dongguan Third People’s Hospital, Affiliated Dongguan Shilong People’s Hospital of Southern Medical University, Dongguan, Guangdong, China; # These authors contributed equally to this work

## Abstract

**Objective(s):**

Rotavirus (RV) is one of the most significant pathogens associated with childhood diarrhoeal deaths worldwide. Elevated cytoplasmic calcium is required for RV replication, but the underlying mechanisms responsible for calcium influx remain poorly understood. The Calcium-sensing receptor (CaSR) is an important Ca^2+^ sensor that regulates the transport of Ca^2+^ into or out of the extracellular space by affecting the status of Ca^2+^ ion channels on the membrane of cells. Currently, the function of CaSR in RV replication is unclear.

**Materials and Methods:**

We evaluated the mRNA and protein levels of CaSR in RV-infected cells using qRT-PCR and Western blotting, respectively. Furthermore, we silenced or overexpressed CaSR in Caco-2 cells using siRNA or a CaSR gene contained adenovirus (Adv-CaSR). qRT-PCR, plaque assay, and Western blotting were used to determine the synthesis of virus genomic RNA, production of progeny virion, and the levels of viral proteins. The content of Ca^2+^ in cells was observed under confocal microscopy.

**Results:**

Compared with control cells, the RV-infected cells presented significantly decreased CaSR expression. Moreover, adenoviral-mediated over-expression or induction of CaSR by R568 greatly inhibited the RV RNA synthesis, protein expression, and formation of viroplasm plaques, thereby suppressing RV replication. In contrast, CaSR-silenced cells exhibited significantly enhanced RV replication. Compared with the Adv-Control group, the concentration of cytosolic Ca^2+^ significantly decreased in the Adv-CaSR group.

**Conclusion:**

These findings demonstrated that CaSR is a potential target for inhibition of RV replication. Therefore, enhancing the expression of CaSR might protect hosts from RV infections.

## Introduction

Rotavirus (RV) is one of the most significant pathogens associated with childhood diarrhoeal deaths worldwide (1). As a non-enveloped double-stranded RNA (dsRNA) virus, RV can lead to serious cell death and dysfunction of ionic homeostasis *in vivo* (2). The homeostasis of Ca^2+^ is significantly associated with many pathological processes, including viral infections. To establish a friendly environment in cells to favor assembly and replication, the disruption of host Ca^2+^ homeostasis is the first step after many virus infections (3). Consistently, RV dysregulates calcium homeostasis by activating Ca^2+ ^influx channels on the membrane of cells and decreasing endoplasmic reticulum (ER) stores (4, 5). The ER and extracellular Ca^2+^ are considered the major source of the increased cytoplasmic Ca^2+^ concentration ([Ca^2+^]_cyto_). Since the Ca^2+^ in the extracellular pool is much greater than that stored in ER, most [Ca^2+^]_cyto_ after RV infections is derived from the extracellular pool. The rotaviral nonstructural protein 4 (NSP4) is required for RV entry, transcription of viral genes, morphogenesis, lysis of infected cells, release of the viral particles, and the distant action of viral proteins, and can be rapidly induced by RV infections (2). Several studies have investigated the mechanisms of [Ca^2+^]_cyto_ elevations. Moreover, inhibiting the increase of intracellular Ca^2+ ^might be beneficial to inhibit virus replication and reproduction in RV-infected cells.

The calcium-sensing receptor (CaSR) is a G protein-coupled receptor identified in multiple tissues, such as the kidney, parathyroid glands, and cardiovascular and the entire digestive system (6). The importance of CaSR in the maintenance of systemic calcium homeostasis, fluid balance, and osmotic regulation has been demonstrated by several previous studies (7-9). As a key regulator of Ca^2+^, CaSR can regulate Ca^2+ ^release from ER, as well as control the status of Ca^2+^ ion channels on the membrane of cells, thereby controlling Ca^2+ ^transport between the cytoplasm and extracellular space (10). Our previous research showed that the basolateral membrane of colon epithelial cells from the RV-induced diarrhea group exhibited significantly decreased CaSR expression at the transcription and translation levels (11). We also found that down-regulation of CaSR is associated with diarrhea severity. Currently, the biological function of CaSR during RV infections has not been completely clarified. Therefore, in the present study, we investigated the involvement of CaSR in RV infections. We also showed that RV infections can significantly suppress the expression of CaSR in cells. Furthermore, enhanced expression of CaSR in cells can dramatically impair RV replication. In contrast, inhibition of CaSR expression promoted RV replication. Our current results provided novel insights into the cellular mechanisms of CaSR contributing to pathogenesis and RV replication. Additionally, activation of CaSR might be a potent therapeutic target to reduce RV infections.

## Materials and Methods

***Cells and viruses***

Monkey kidney MA104 (ATCC number: CRL-2378), Human intestinal HT-29 (ATCC number: HTB-38), SW620 (ATCC number: CCL-227), and Caco-2 (ATCC number: HTB-37) cells were obtained from the American Type Culture Collection (ATCC, USA). Cells were routinely cultured in FBS (10%, Gibco), penicillin (100 U/ml), and streptomycin (100 mg/ml) containing Dulbecco’s-modified Eagle’s medium (DMEM, Gibco, USA). Dr. Haiyang He (Institute of Immunology, Third Military Medical University, China) kindly gifted the RV Wa (human strain; G1P [8]). After pre-activation using crystallized trypsin (10 μg/ml), MA104 cells were used to inoculate and propagate the RV at a multiplicity of infection (MOI) of 2. Cells were harvested after 48 hr of inoculation and cytopathic effect (CPE) > 95 %. The CsCl isopycnic gradients were applied for RV purification as previously described (12). 

***Reagents and antibodies***

We obtained CaSR agonist calcimimetic NPS R568 from Sigma-Aldrich. The anti-CaSR antibody was provided by Abcam Biotechnologies. Anti-VP6, anti-NSP4, and anti-GAPDH antibodies were provided by Santa Cruz Biotechnologies. The goat anti-rabbit/mouse IgG (H+L)/TRITC and FITC-conjugated goat anti-mouse IgG were purchased from Invitrogen.

***RV plaque assay***

Plaque assays were conducted using Caco-2 cells to access the virus titer in the supernatant of the culture (13). Briefly, after culture for the indicated times, Caco-2 cells (2 ×10^6 ^cells per well) were co-cultured with 10-fold series diluted RV (400 μl) at 37 °C for 1 hr. After removing the culture medium, we covered the plate with trypsin (2.5 μl/ml), sodium bicarbonate (1%), kanamycin (0.1 mg/ml), gentamicin (0.05 mg/ml), HEPES (15 mM, PH 7.7), and L-glutamine (2 mM) containing 1% agarose DMEM. Then, after 72 hr of incubation at 37 °C and 2 hr of fixation with 10% formaldehyde, crystal violet staining was conducted to visualize the plaques. Plaque-forming units per ml (PFU/ml) were used to present the virus titer. 

***Knockdown of CaSR using siRNA***

Lipofectamine 2000 (Invitrogen) was used to perform the CaSR siRNA (si-CaSR) transfection into Caco-2 cells. The cells transfected with non-targeting siRNA (siNC) were used as the negative control. Then, the RV (MOI = 1) was used to infect the cells after 48 hr of incubation. Finally, we collected the cells and culture supernatants at 12 and 24 hr after RV infection for further analysis.

***Generation, collection, and purification of recombinant adenoviruses***

For cloning of the Human CaSR gene, we used the shuttle vector pLEX-MCS-RGN-CMV (Weizhen Biological Company, Shandong, China), plasmid pAdEasy-1, and *E. coli *BJ5183. For selection and confirmation of the recombinants, kanamycin and restriction endonuclease analyses were carried out, respectively. After digestion using Pac I and transfection, we collected the recombinant adenovirus generated in Caco-2 cells. Next, after 3-time passages in Caco-2 cells, the collected virus was purified using CsCl to obtain a high-titer viral stock. Standard methods were performed to determine the titers of purified viruses (14). 

***qRT-PCR***

After RNA extraction from cells and RV with TRIzol (Invitrogen) and PureLink^®^ Viral RNA/DNA Mini Kit (Invitrogen), respectively, we performed qRT-PCR to examine the relative transcripts of target genes using the Real-Time SYBR™ Green PCR Master Mix kit (Applied Biosystems) on the ABI7500 Real-Time PCR System. GAPDH was used as the housekeeping gene to normalize target gene levels. The sequences of primers for qRT-PCR are presented in Table 1. All qRT-PCR experiments were conducted in triplicates. 

***Western blotting***

After lysis with RIPA buffer (Beyotime Biotechnology) and quantification with the BCA protein assay kit (Thermo Scientific), proteins were separated and transferred with 10% SDS-PAGE and PVDF membrane, respectively. After 2 hr of blocking using non-fat milk (2.5%) and overnight incubation with different antibodies, the protein bands were incubated with indicated secondary antibodies. Finally, after 3-time washing with PBST, the signals were visualized and reported by the Odyssey Infrared Imaging System (LI-COR Biotechnology). 

***Measurement of [Ca*** ^2+^ ***]***_cyto_

The Fluo-3/AM fluorescence assay was used to examine the levels of [Ca^2+^]_cyto_. Briefly, after 1 hr of incubation with Fluo-3/AM (5 μM) and Pluronic F-127 (0.1%, Sigma) containing HBSS (400 μl) at 37 °C and two times washing using Ca^2+^-free HBSS, a confocal microscope (Leica SP8) was used to visualize and record the fluorescent intensity of Fluo-3/AM in differentially treated cells. 

***Statistical analyses***

Values are presented as means ± standard deviations (SD). For comparisons among more than two groups, one-way ANOVA was performed. To compare the two groups, a two-tailed Student’s t-test was applied. *P*<0.05 was considered statistically significant. GraphPad software (Version 5.01) was used for analyses and to generate the graphs. 

## Results

***RV-infected cells have decreased expression of CaSR***

**Figure 1:**
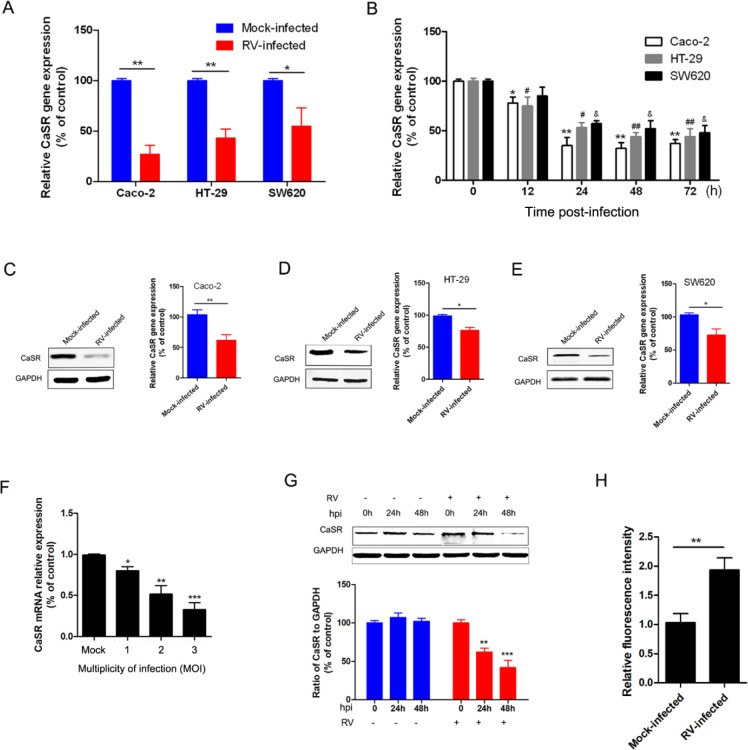
mRNA and protein expression of CaSR in RV-infected cells. (A) Caco-2, HT-29, and SW620 cells were infected with RV-Wa strains at an MOI of 2 for 12 hr. The expression of CaSR was analyzed using qRT-PCR and compared with mock-infected cells (control). (B) mRNA expression of CaSR was analyzed using qRT-PCR at 12, 24, 48, and 72 hr. (C-E) Expression of CaSR proteins was detected in RV-infected Caco-2, HT-29, and SW620 cells by using western blotting and were corrected according to the levels of GAPDH. (F) Caco-2 cells were infected with RV-Wa at the indicated multiplicity of infection (MOI) for 24 hr, then the mRNA expression of CaSR was analyzed by qRT-PCR. (G) Caco-2 cells were infected with RV-Wa, and cell lysates were collected at indicated time points (0, 24, and 48 hpi). The expression levels of CaSR were determined by using western blot analysis. GAPDH was used as a loading control. (H) Cells were inoculated with RV-Wa for 12 hr. The relative fluorescence intensity values represent cytosolic Ca^2+^ content. Each column is presented as the mean ± standard deviation of three independent experiments

To determine the expression of CaSR, qRT-PCR and Western blot analyses were conducted in RV-infected Caco-2, HT-29, and SW620 cells at the transcriptional and translational levels, respectively. First, the mRNA and protein levels of CaSR were down-regulated in RV-infected SW680, HT-29, and Caco-2 cells (Figure 1A-E). Additionally, Caco-2 cells exhibited significantly reduced CaSR expression after RV infection with an MOI and time-dependent pattern (Figure 1F-G). Compared with cells infected with the mock virus, RV-infected cells presented significantly high fluorescent intensity (Figure 1H), suggesting the enhancement of RV infections with increasing [Ca^2+^]_cyto_. 

***Overexpression of CaSR inhibits RV replication***

**Figure 2:**
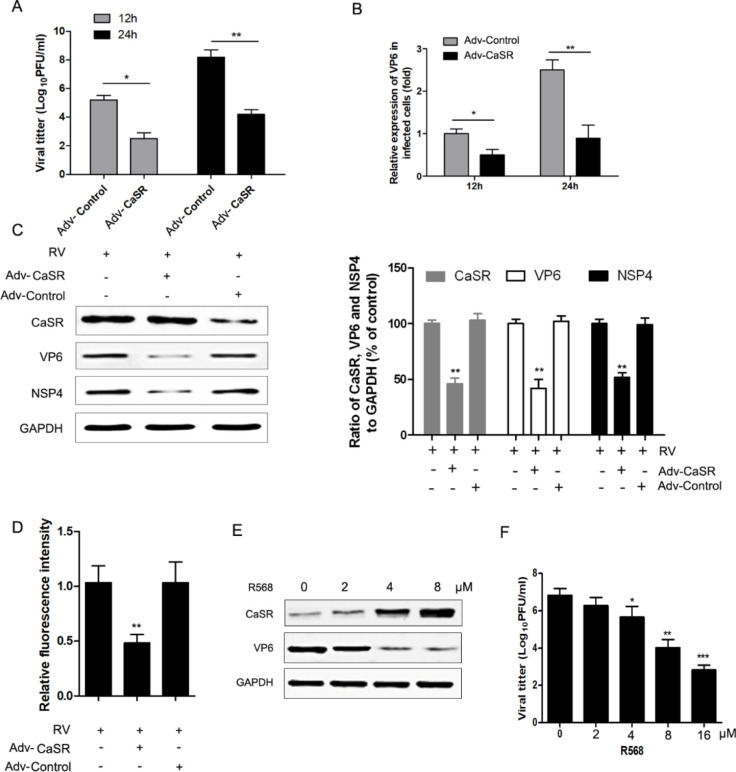
Over-expression of CaSR inhibited RV replication. Caco-2 cells transfected with Adv-CaSR and Adv-control were infected with RV Wa strains at an MOI of 2 for 12 and 24 hr. (A) Virus titers were determined using a plaque assay. (B) Expressions of viral VP6 from Wa strains were determined by qRT-PCR. (C) Expression of viral protein NSP4, VP6, and CaSR was determined by western blot. Western blots were analyzed by Quantity One software (version 4), and the statistical analysis is shown. (D) Relative fluorescence intensity values represent cytosolic Ca^2+ ^content. Each column is presented as the mean ± standard deviation of three independent experiments. (E) Caco-2 cells were incubated for 24 hr with various concentrations of R568 (0-20 μM), a CaSR-specific agonist. Subsequently, virus titers were determined using a plaque assay. (F) Caco-2 cells were incubated for 24 hr with various concentrations of R568, then the expression of viral protein VP6 and CaSR was determined by western blot. The results of 3 independent experiments are presented as the mean±standard deviation (SD). *P<0.05 and **P<0.01

Compared with cells transfected with the control virus (Adv-Control), cells transfected with Adv-CaSR exhibited dramatically reduced viral titers and impaired synthesis of RV RNA (Figure 2A-B). Moreover, CaSR overexpression led to a significant reduction in the NSP4 and VP6 levels of RV (Figure 2C). Additionally, compared with the Adv-Control group, the concentration of cytosolic Ca^2+^ decreased in the Adv-CaSR group (Figure 2D). Similarly, inhibition of CaSR using calcimimetic NPS R568 significantly reduced the viral titers and VP6 expression in a dosage-dependent manner (Figure 2E-F). 

***Inhibition of CaSR expression promotes RV replication***

**Figure 3:**
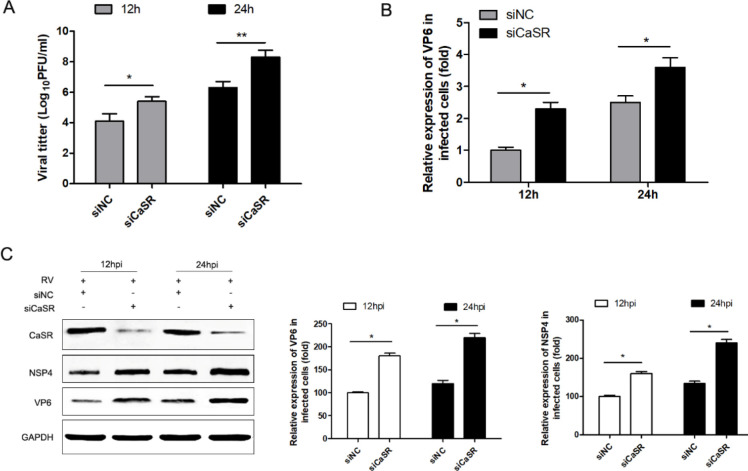
CaSR knockdown promoted RV replication in Caco-2 cells. Caco-2 cells were transfected with non-targeting control (NC) siRNA or siRNA (100 nM) targeting CaSR for 12 hr followed by infection with Wa at an MOI of 2 for 12 and 24 hr. (A) Wa-RV virus titers were determined using a plaque assay. (B) Levels of expression of RNA of RV were determined by using qRT-PCR. (C) Expression of viral protein NSP4, VP6, and CaSR was determined by western blot. Western blots were analyzed by Quantity One software (version 4), and the statistical analysis is shown. The results of 3 independent experiments are presented as the mean ± standard deviation (SD). *P<0.05 and **P<0.01

The above results indicated that CaSR overexpression can significantly suppress the replication of RV. To further confirm these results, we silenced CaSR in Caco-2 cells, then infected them with RV. The results showed that the amount of progeny virus and the synthesis of RV RNA were dramatically enhanced in siCaSR-transfected cells at 12 and 24 hr compared with the negative control (Figure 3A-B). Consistently, compared with siNC-transfected cells infected with RV, siCaSR transfection resulted in a significant increase in NSP4 and VP6 protein levels. 

***Deficient expression of CaSR promotes RV***

**Figure 4:**
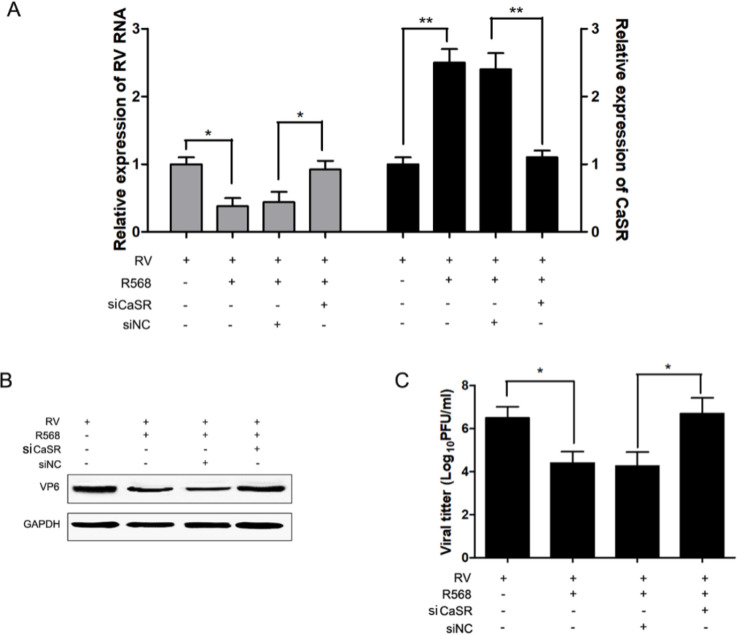
Knockdown of CaSR reversed CaSR-inhibition of RV replication. Caco-2 cells were transfected with a CaSR siRNA or NC siRNA (100 nM) for 12 hr and then infected with RV at an MOI of 1, followed by treatment with or without 20 nM of R568 for 24 hr. (A) RV RNA and CaSR expression were measured by qRT-PCR. (B) RV VP6 protein expression was evaluated by using western blotting. (C) The cells were then harvested for detection and the infectious virus titers in cell supernatants were evaluated by using plaque assays

Furthermore, we treated Caco-2 cells in which CaSR expression was silenced or not using a CaSR agonist to confirm its significance for RV replication. We observed that the suppressive effect of NPS R568 on RV replication was dramatically restored by CaSR silencing in Caco-2 cells (Figure 4A). The restored viral replication and protein production were also significantly observed in the CaSR deficient cells in the presence of R568 (Figure 4B-C). 

**Table 1:** Primers used in this study for Real-Time PCR

Primer name	Orientation	Sequences (5’-3’)
RV	Sense	TGGCTTCGCTCATTTATAGACA
	Antisense	ATTTCGGACCATTTATAACC
GAPDH	Sense	GTCAACGGATTTGGTCGTATTG
	Antisense	TGGAAGATGGTGATGGGATTT
VP-6	Sense	CAGTGATTCTCAGGCCGAATA
	Antisense	GGCGAGTACAGACTCACAAA
CaSR	Sense	AGGCCGGAGTCTGTGGAATGTA
	Antisense	CAGCGTCAAGTTGGGAAGAAGG
NSP4	Sense	GGCAATTAAAAGTCCAGTTATGG
	Antisense	GGGTCCTTATCAGTTTGATCAG

## Discussion

Acute diarrhea caused by RV infections is considered a serious public health problem worldwide, resulting in almost 500,000 deaths each year. However, drugs that can effectively protect patients from RV infections, as well as RV-mediated gastroenteritis, are not available. Thus, the development of new pharmacological drugs for effective anti-RV therapies is critical. All viruses, including RV, need to change the environment of the host to favor their infection, replication, and subsequent spread. Hence, the viruses had to develop the potential to modify, change, or control the signaling pathways of host cells, to finally trigger changes in cell apoptotic, cell survival, homeostatic, or survival status to let them escape or survive the host’s immune attack. To understand the interactions between virus and host cells, and the pathogenesis of viral infection-caused diseases, identifying the key mediator in host cells for the regulation of viral replication is urgently required. 

The homeostasis of Ca^2+^ in cells is significantly associated with many biological functions, including viral infections. Many studies have suggested that viruses can significantly disrupt the Ca^2+^ homeostasis of infected host cells to favor their replication and growth. Before entering cells, the extracellular Ca^2+ ^plays a significant role in the stabilization of the viral capsid. However, low levels of Ca^2+^ in cells are important to the degradation of viral capsid and subsequent activation of transcriptase after the virus entry into cells. The Ca^2+^ homeostasis modified by viral proteins can promote virus morphogenesis and cause cell death (15). However, the mechanisms by which viroporin activity in the ER activates the transport of Ca^2+^ across the PM remains unknown. In RV-induced diarrhea, it is believed that NSP4 acts as a secreted viral enterotoxin that can increase the calcium concentration in cells by promoting calcium release from ER, interacting with integrin receptor α1ß2, or binding and releasing with galanin (16-18). The calcium-activated chloride channel(s) (CaCCs) can be activated by increased calcium in the cytoplasm, thereby inducing the secretion of chloride secondary sodium and water (19). 

CaSR has been reported to be expressed on the GI tract and inflammatory cells and to significantly participate in several physiological and pathological processes. The tight association between abnormal CaSR expression and the development of pathological diarrhea has been previously observed by many studies (20, 21). Our previous research indicated that the mRNA and protein levels of CaSR were significantly attenuated in the basolateral membrane of colon epithelial cells in the RV-induced diarrhea group (11). We also found that down-regulation of CaSR is associated with the severity of diarrhea. Currently, the biological function of CaSR during RV infections remains unclear. Herein, we investigated the involvement of CaSR in RV infections and demonstrated that the expression of CaSR was down-regulated in RV-infected cells. Moreover, we showed that overexpression of CaSR greatly inhibited RV replication by suppressing the synthesis of RV RNA, protein expression, and formation of viral plaques. Additionally, compared with the Adv-Control group, the concentration of cytosolic Ca^2+ ^decreased in the Adv-CaSR group. Thus, RV infections can modify Ca^2+^ homeostasis by regulating the expression of CaSR. Using gene overexpression and siRNA-mediated gene silencing assays, we further demonstrated the specific antiviral effect of CaSR in Caco-2 cells. The CaSR silenced cells exhibited significantly enhanced synthesis of RV mRNA, expression of viral proteins, and production of progeny virus, suggesting the significant role of CaSR in RV replication. 

Furthermore, we also demonstrated that the CaSR agonist, calcimimetic NPS R568, greatly inhibited RV replication by suppressing the expression of RV proteins and the formation of viral plaques. Additionally, R568 significantly suppressed RV replications, which were reversed by pretreatment with siCaSR. These observations confirmed that enhancing the expression of CaSR can effectively impair RV replication in cells, suggesting the importance of CaSR to protect patients from RV infections in the clinic. 

## Conclusion

We showed that enhancing the expression of CaSR can suppress RV infection, replication, and spread. These results indicate the potential of using CaSR agonist as a novel treatment to prevent RV infections. Additionally, further evaluation and characterization of CaSR agonists on RV-induced diarrhea are warranted. 

## Authors’ Contributions

HH and DL Participated in the design of this study, carried out the experiments, performed the statistical analysis, and drafted the manuscript. BH and YC performed the experiments and analyzed the data. RP and GZ Analyzed and interpreted data, and supervised, directed, and submitted the paper. All authors have read and approved the final version of the manuscript.

## Conflicts of Interest

The authors declare that they have no competing interests.
